# Usefulness of INTELLiVENT-ASV for postoperative ventilator-associated pneumonia: a case report

**DOI:** 10.1186/s40981-019-0262-x

**Published:** 2019-06-27

**Authors:** Takayuki Taira, Tatsuya Fuchigami, Misuzu Hayashi, Kouta Kamizato, Kouji Teruya, Manabu Kakinohana

**Affiliations:** Department of Anesthesiology, Ryukyu University School of Medicine Nishihara, Nakagami-gun, Nishihara-cho, Uehara, Okinawa, 207 Japan

**Keywords:** Artificial respiration, INTELLiVENT® iASV, Ventilator-associated pneumonia

## Abstract

**Background:**

INTELLiVENT®-ASV (iASV) is a respiration mode on the Hamilton G5. The ventilator uses a closed-loop mechanism that automatically adjusts settings related to oxygenation and ventilation.

**Case presentation:**

A 47-year-old man underwent reconstruction surgery with free musculocutaneous flap for tongue resection. After surgery, the patient entered the ICU, and the iASV, which automatically changed only the percent minute volume (%MV) in respiration mode, was selected. On the second day, ventilator-associated pneumonia (VAP) was diagnosed, and the antibiotic treatment was changed. Using the settings of the iASV, automated FiO2 and positive end-expiratory pressure (PEEP) control were added to the ventilator mode. The patient’s oxygenation was improved.

**Conclusions:**

In a patient who developed VAP after surgery, ventilation was continued using iASV, and automated changes in PEEP and FiO2 settings were successfully made according to the open lung strategy, under short-staffed circumstances.

## Background

INTELLiVENT®-ASV (iASV) is a mechanical ventilation mode on the Hamilton G5 that uses a closed-loop mechanism; it allows monitoring of the transcutaneous arterial blood oxygen saturation (SpO_2_) and end-tidal carbon dioxide tension (ETCO_2_) in real time. If the operator sets the target value of SpO_2_ and the ETCO_2_, the ventilator automatically adjusts the settings related to oxygenation (FiO_2_ and PEEP) and ventilation (target minute ventilation, tidal volume, and ventilation frequency). iASV is based on adaptive support ventilation (ASV®), which guarantees minute ventilation.

ASV differs from the conventional mechanical ventilation mode in that the operator sets the target minute ventilation volume (percent minute volume, %MV), instead of the tidal volume (VT) and respiratory rate (*f*).

A combination of VT and *f* that achieves %MV is automatically set from the respiratory algorithm. It can be applied to patients in all states of ventilation, from assist control to spontaneous, and it has been reported to be particularly useful in ventilator weaning [1, 2].

During ASV ventilation, a safe range is automatically set based on the values of tidal volume and respiratory rate measurements, such as airway pressure and flow rate, which are obtained from the respiratory circuit. This avoids volume- and pressure-related damage that can occur with excessive tidal volume, dead-space ventilation, which can be problematic with an inappropriate tidal volume and inappropriate ventilation, for instance with apnea, as well as auto-positive end-expiratory pressure (PEEP), which can arise with an excessive number of breaths [3]. It has also been reported that iASV can ensure better optimal lung protective ventilation than other conventional ventilation modes and ASV [4].

In this case report, we describe our experience with a case in which the patient, who was on artificial respiration with deep sedation after surgery, became hypoxic due to ventilator-associated pneumonia (VAP) and for whom ventilator management by iASV was particularly useful.

## Case presentation

**Fig. 1:**
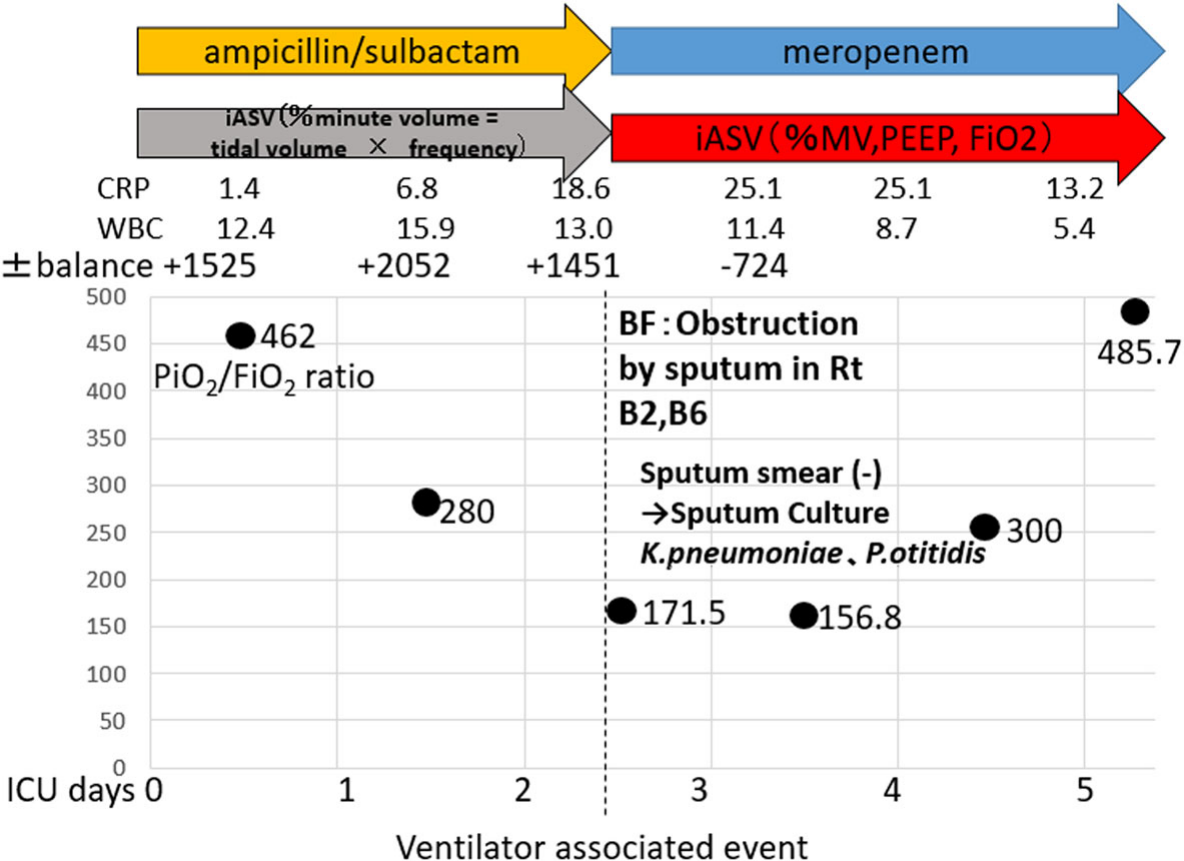
Hospitalization process. At the time of entering the intensive care unit, the patient’s PaO_2_/FiO_2_ ratio was 462(black point), which indicated a good oxygenation capacity; this decreased to 171.5 at the time of diagnosis of ventilator-associated pneumonia

**Fig. 2:**
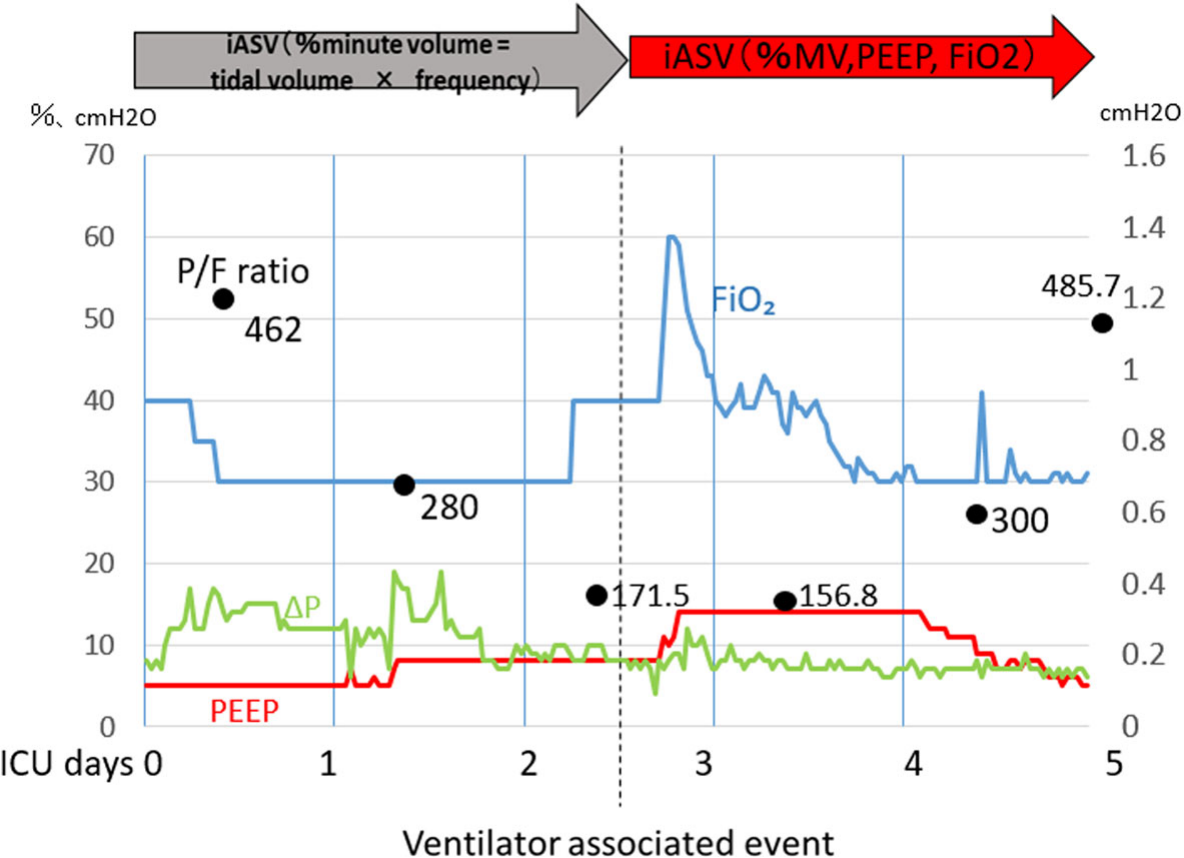
Progress of iASV. PEEP and FiO_2_ were selected as additional automatic settings in iASV, over and above %MV, and continuous ventilation. Using the automatic setting of iASV, PEEP was set at 14 mmHg against the decrease in oxygenation capacity, and FiO_2_ and PEEP gradually increased concurrently to reach a FiO_2_ value of 0.6. FiO_2_ fell notably after recovery of oxygenation capacity, while PEEP gradually decreased after FiO_2_ reached 0.3. During this time, the driving pressure (Δ*P*) obtained by subtracting PEEP from the intake plateau pressure was below 10 cm H_2_O

A 47-year-old man (body weight 62.1 kg and height 166.8 cm) was reported in our hospital with a past medical history of tongue cancer that was operable. The preoperative spirometry test, electrocardiography, and transthoracic echocardiogram were normal. He is a non-smoker with no history of cerebrovascular disease. In his thirties, he had undergone resection of the mandibular area with plate reconstruction for treatment of tongue cancer; however, the site became infected and the plate was removed 8 months before presentation. The patient returned for reoperation involving a free abdominal muscle flap with vascular anastomosis and partial mandibular resection with tracheostomy. After surgery, the patient was in the ICU for deep sedation and ventilator management for 48 h, until the vascular anastomosis stabilized. Initially, the iASV ventilator mode was selected to change only the percentage minute volume (%MV) automatically. On the postoperative day 3, the patient’s oxygenation worsened, and purulent sputum increased; fiberoptic suctioning of sputum was performed once along with sputum culturing. Although no significant organism was detected with sputum Gram staining, *Klebsiella pneumoniae* and *Pseudomonas otitidis* were detected in the sputum culture test. We therefore arrived at a diagnosis of VAP and changed the patient’s antibiotic from ampicillin/sulbactam 1.5 g per 6 h to meropenem 0.5 g per 8 h. At the time of entering the ICU, his PaO_2_/FiO_2_ ratio was 462, which indicated a good oxygenation capacity; this decreased to 171.5 at the time of VAP diagnosis. At the same time, the other patient entered the ICU with a severe respiratory emergency. We continued the iASV mode as we judged that the respiratory condition could be improved. PEEP and FiO_2_ were selected as additional automatic settings in iASV, %MV was automatically set, and the delta *P* (Δ*P*) approximating the driving pressure did not exceed 10 cm H2O. (Figs. 1 and 2).

Arterial blood gas (PaO2, PaCO2, and pH) was measured every 6 h to confirm that his respiratory condition was within the expected range.

### Progress after VAP diagnosis

Once the patient’s oxygenation capacity started improving, the INTELLiVENT®-ASV started to show a decline in FiO2, first until it reached 0.3, and then reduced PEEP from 14 to 5 cm H2O based on the lung protective ventilation strategy. Two days after changing the antibiotics to treat VAP, his WBC, CRP, and the sputum secretion from the lungs decreased, indicating resolution of VAP. Therefore, mechanical ventilation was successfully withdrawn. During this time, the Δ*P* obtained by subtracting PEEP from the inspiratory plateau pressure was kept below 10 cm H_2_O (Fig. 2).

We used dexmedetomidine (0.2*γ* to 0.5*γ*) and propofol (1 mg/kg/h to 3 mg/kg/h) for sedation during his admission in ICU. We continued the same sedation dosage after VAP diagnosis, and after stopping ventilator usage, the propofol administration was also stopped. Dexmedetomidine was continued after extubation at night for the purpose of sleeping.

Two days after stopping ventilator usage, the patient’s respiratory condition was stable and he was transferred to the general ward.

## Discussion and conclusions

The patient developed VAP during deep sedation, following ventilation, to allow recovery for 48 h after surgery. The patient’s oxygenation also decreased. Thus, we used the iASV mode as there was a shortage of manpower in the hospital. Antibiotic treatment was effective, and the respiratory condition of the patient improved.

Amato et al. reported improvement of outcomes using low tidal volume ventilation (LTVV) [5]; therefore, the importance of limiting the tidal volume (VT), as a lung protective strategy, has been emphasized. Several randomized control trials on LTVV have been conducted to date. In a systematic review of these reports, the safety of LTVV and the risk of an excessive plateau pressure have been pointed out [6, 7].

The Berlin definition [8], which was announced as an alternative to the definition of acute respiratory distress syndrome (ARDS) set out by the American–European Consensus Conference and used since 1992, includes the evaluation of oxygenation performance by adding a certain PEEP load to the diagnostic criteria, suggesting the importance of an appropriate PEEP load. Bellani et al. [9] investigated the real-life practice of treating ARDS in an ICU in 459 countries across 5 continents using the Berlin definition. In total, 2377 patients were diagnosed with ARDS within 48 h and were intubated; however, in one-third of these cases, a ventilation limit of VT < 8 ml/kg was not used, and the plateau pressure measurement remained at 40.1%. In addition, PEEP was set at less than 12 cm H_2_O in 82.6% of cases; the PEEP setting did not reach this level, even with inhalation of a high concentration of oxygen. Therefore, ARDS patients were not considered to have received adequate ventilation. Furthermore, Bellani et al. also predicted a shortage of medical staff to cope with the increase in ventilation patients [9], and thus, that it would become challenging to ensure appropriate ventilation management on a continuous basis.

It has been reported that iASV can facilitate more appropriate ventilation management [10] than that which is achieved with the conventional facility protocol, without burdening the medical personnel [11]. The closed-loop mechanism of the iASV reduces the work of medical staff, and thus, it is expected that this treatment will become a standard of treatment. Additionally, iASV can improve oxygenation capacity while maintaining lung protective ventilation [12] comparable to conventional management. The protocols proposed in the ARMA study [13] and the ALVEOLI study [14] were incorporated in the closed-loop setting algorithms related to oxygenation by the INTELLiVENT (i.e., implementation of FiO_2_ and PEEP settings), based on an open lung strategy, in the presented case.

To date, no report has detailed the progression of automatically changing the ventilation settings in iASV for patients who developed VAP during the observation period, as in our case. In this case, we noted that FiO_2_ and PEEP gradually increased while the oxygenation capacity declined; there was a predominant decline in FiO_2_ after recovery of oxygenation capacity and that PEEP gradually decreased after FiO_2_ reached 0.3. During this period, the tidal volume could be limited to 8–10 ml kg, and the plateau pressure could be limited to 15 cm H_2_O or less. In this case, PEEP increased automatically to up to 14 cm H_2_O, but in cases of sepsis from VAP and further septic shock, an automatic increment in PEEP leads to significant circulatory suppression, possibly due to impaired venous return.

iASV has a feedback mechanism that evaluates circulatory dynamics from respiratory fluctuation of a pulse oximeter waveform called the heart lung index (HLI), and the PEEP was changed automatically by the ventilator. This safety mechanism did not operate in our case because of change in the circulation dynamics. In this case, it is possible that fluctuation in the circulation dynamics due to the sepsis accompanying VAP and duplication of the circulating blood volume by transfusion after the surgery functioned synergistically to maintain the cardiac function. This may have prevented circulation suppression due to the increase in PEEP.

The use of iASV has certain limitations. It is unknown whether iASV can always be used in all patients with VAP or whether the HLI is effective. In iASV, we checked arterial blood gas analysis as previously mentioned, and continuous observation of respiration and circulation remains necessary. Furthermore, iASV automatically increases the set values of FiO_2_ and PEEP when the oxygen levels are low, which may delay the awareness of ventilator-associated events by the medical staff; therefore, warning alerts at this stage and improvement of the display function may be necessary.

In summary, we experienced a case in which the use of iASV was effective following the onset of VAP after surgery. While the closed-loop mechanism of iASV may contribute to the implementation of a lung protective strategy and reduce the labor of medical staff, it still needs improvement. Furthermore, when using iASV, it is also important to understand the mode of ventilation and to observe respiration and circulation conventionally.

## Data Availability

Available upon request.

## Abbreviations

ARDS: Acute respiratory distress syndrome
ASV: Adaptive support ventilation
BF: Bronchofiber
ETCO2: End-tidal carbon dioxide concentration
FiO2: Fraction of inspiratory oxygen
HLI: Heart lung index
iASV: INTELLiVENT®-ASV
ICU: Intensive care unit
LTVV: Low tidal volume ventilation
MV: Minute volume
PEEP: Positive end-expiratory pressure
SpO2: Percutaneous arterial blood oxygen saturation
VAE: Ventilator-associated events
VAP: Ventilator-associated pneumonia
VT: Tidal volume
